# Oral immunotherapy in clinical practice

**DOI:** 10.1186/1824-7288-41-S2-A2

**Published:** 2015-09-30

**Authors:** Stefania Arasi, Lucia Caminiti, Giuseppe Crisafulli, Giovanni B Pajno

**Affiliations:** 1 Department of Pediatrics-Allergy Unit, University of Messina, Messina, 98121, Italy

## Introduction

Food avoidance is still considered the gold-standard treatment for food allergy (FA). However, recently it has been consolidating the theory that, where possible, the food intake might facilitate the induction of desensitization and tolerance, resulting more effective and safer -if we consider the possibility of accidental ingestion- than a strict avoidance diet [1].

Furthermore, specific oral immunotherapy (OIT) is the unique active treatment for IgE-mediated FAs [1].

## Efficacy

Studies have shown short-term efficacy: namely theOIT-ability to induce desensitization (loss of responsiveness to food-antigen during continuous assumption of OIT-doses) [2-5]. Conversely, data are still insufficient and controversial regarding long-term efficacy: namely, the achievementof tolerance(configured when the offending food is removed from the diet and, if reintroduced, does not elicit any adversereaction) [2-5].

Recent data have shown that a key role in the induction of tolerance by OIT is played by a subgroup of regulatory T cells known as “induced T reg” (iTreg) [6,7]. Epigenetic studies in vivo assessthatOIT determines hypomethylation of FOXP3 protein in specific antigen-iTreg only in individuals tolerant. Therefore, changes in the DNA of iTreg - specific antigen may predict the development of a state of clinical immune tolerance during OIT [8].

## Safety

About safety, allergic reactions occurred in the majority of patients treated. They are primarily mild reactions, severe in about 4%; however, no threatening-life events nor death has been reported [9]. Frequency and severity of side effects seem reduced in protocols involving more gradual dose increases [1].

Careful clinical monitoring by qualified personnel in a medical environment is essential. Consequently, OIT costs are high.

In all immunotherapy treatments, safety is of paramount importance. Therefore, when severe adverse reactions occur, OIT should be stopped and revaluated.

## Possible approches improving OIT

Several efforts have been made to perfect OIT: e.g.antigens with reduced allergenicity and adjuvants inducing faster immune responses of “protection”. Particularly promising is OIT associated with omalizumab, anti-IgE antibody, resulting safe and able to accelerate the desensitization compared to traditional protocols [10-12].

## Conclusions

Further researches are needed in order to verify the degree of long-term safety as well as the long-term efficacy of OIT.

Given the interest and the celerity of development of this field of research in the recent years, we are confident in the near achievement of a personalized treatment, planned, safe and effective, to be used in clinical practice.
